# Growth and toxin production of phomopsin A and ochratoxin A forming fungi under different storage conditions in a pea (*Pisum sativum*) model system

**DOI:** 10.1007/s12550-021-00446-8

**Published:** 2021-12-18

**Authors:** Birgitta Maria Kunz, Laura Pförtner, Stefan Weigel, Sascha Rohn, Anselm Lehmacher, Ronald Maul

**Affiliations:** 1 Department for Safety in the Food Chain, German Federal Institute for Risk Assessment (BfR), Max-Dohrn-Str. 8-10, 10589 Berlin, Germany; 2 Institute for Food Chemistry, Hamburg School of Food Science (HSFS), University of Hamburg, Grindelallee 117, 20146 Hamburg, Germany; 3 Food Microbiology, Institute for Hygiene and Environment Hamburg, Marckmannstraße 129a, 20539 Hamburg, Germany; 4 Institute for Food Technology and Food Chemistry, Technische Universität Berlin, Gustav-Meyer-Allee 25, 13355 Berlin, Germany; 5 Department Safety and Quality of Milk and Fish Products, Max Rubner-Institut, Federal Research Institute of Nutrition and Food, Hermann-Weigmann-Straße 1, 24103 Kiel, Germany

**Keywords:** *Phomopsis leptostromiformis*, *Diaporthe toxica*, *Aspergillus westerdijkiae*, Storage, LC–MS/MS

## Abstract

Phomopsins are mycotoxins mainly infesting lupines, with phomopsin A (PHOA) being the main mycotoxin. PHOA is produced by *Diaporthe toxica*, formerly assigned as toxigenic *Phomopsis leptostromiformis*, causing infections in lupine plants and harvested seeds. However, *Diaporthe* species may also grow on other grain legumes, similar to *Aspergillus westerdijkiae* as an especially potent ochratoxin A (OTA) producer. Formation of PHOA and OTA was investigated on whole field peas as model system to assess fungal growth and toxin production at adverse storage conditions. Field pea samples were inoculated with the two fungal strains at two water activity (*a*_*w*_) values of 0.94 and 0.98 and three different levels of 30, 50, and 80% relative air humidity.

After 14 days at an *a*_*w*_ value of 0.98, the fungi produced 4.49 to 34.3 mg/kg PHOA and 1.44 to 3.35 g/kg OTA, respectively. Strains of *D. toxica* also tested showed higher PHOA concentrations of 28.3 to 32.4 mg/kg.

*D. toxica* strains did not grow or produce PHOA at an *a*_*w*_ values of 0.94, while *A. westerdijkiae* still showed growth and OTA production.

Elevated water activity has a major impact both on OTA and, even more pronouncedly, on PHOA formation and thus, proper drying and storage of lupins as well as other grain legumes is crucial for product safety.

**Supplementary information:**

The online version contains supplementary material available at 10.1007/s12550-021-00446-8.

## Introduction

Grain legumes, including lupine seeds are globally used for animal feed and human nutrition. Nowadays, an increasing number of vegetarians and vegans consume products made from legumes such as meat substitutes, pasta, or bakery products. Similar to cereal grains, grain legumes may also be contaminated with mycotoxins.

**Fig. 1:**
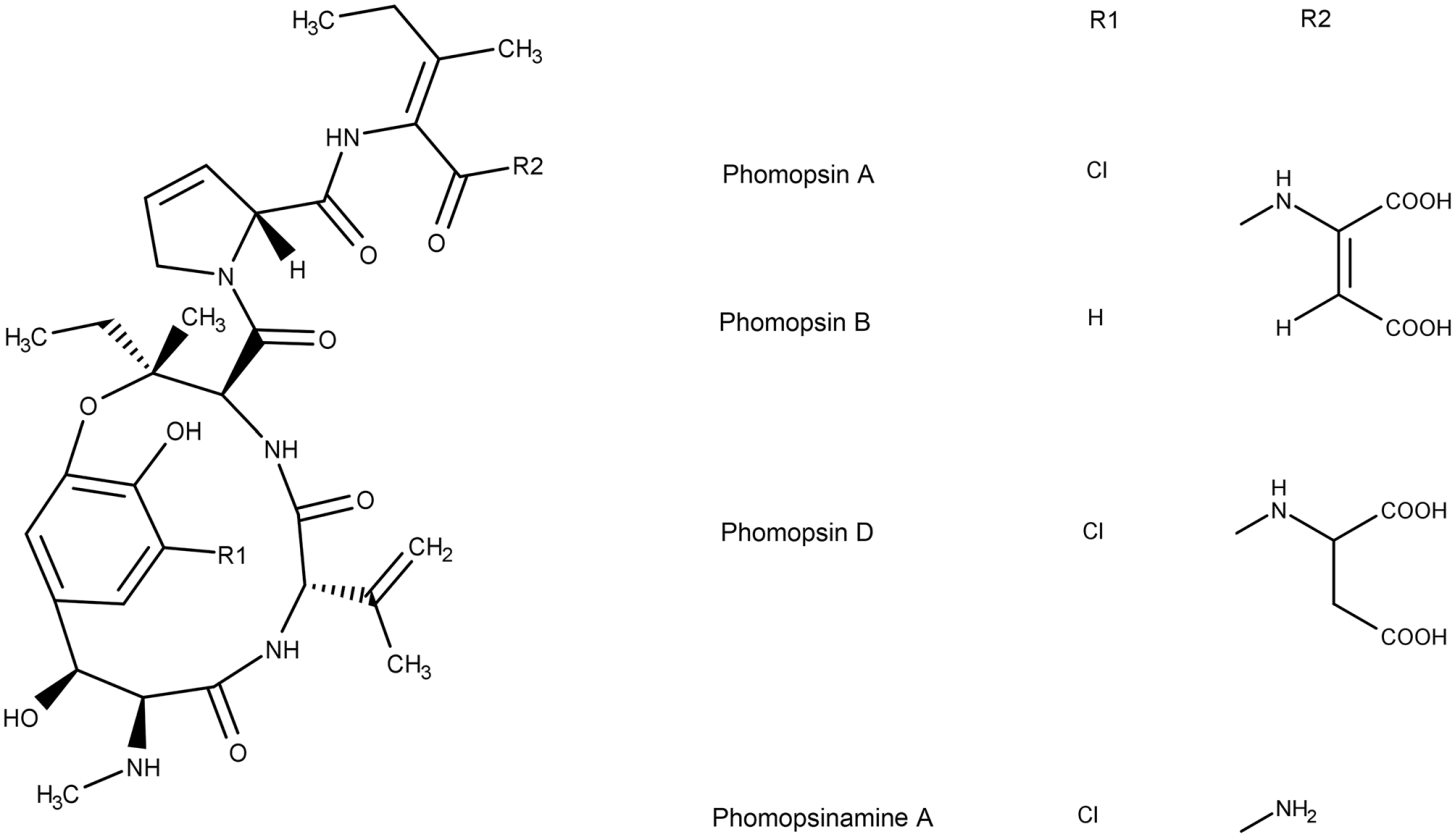
Chemical structures of phomopsins (adapted from Battilani et al. (2011))

When evaluating the contamination status of grain legumes, phomopsin A (PHOA) has to be taken into account. PHOA is the lead toxin of a group of secondary metabolites formed by the mycotoxigenic species *Diaporthe toxica*, globally referred to as *phomopsins*, whose molecular structures are visualized in Fig. 1. They can be assigned to the *emerging mycotoxins*, a group of mycotoxins that might potentially be a risk to consumers, but that is still lacking comprehensive data for conducting a risk assessment. In 2010, the European Food Safety Authority (EFSA) conducted a call for data on phomopsin occurrence (EFSA 2010) and a subsequent risk assessment in 2012 where EFSA called for validated analytical methods (EFSA 2012). Another call for data collection of phomopsins in food and feed was published in the year 2021 (EFSA 2021). Phomopsins are liver toxic causing symptoms such as lupinosis, mainly in grazing sheep (van Warmelo and Marasas 1972; Gardiner 1975). PHOA binds to tubulin and leads to cell cycle arrest, causing cell death in hepatocytes (Battilani et al. 2011) and is cancerogenic in rats (Peterson 1990). *The Food Standards Australia New Zealand* (former *Australia New Zealand Food Authority*, ANZFA) (ANZFA 2001) and EFSA (EFSA 2012) have published risk assessment reports where they stated that data is still lacking. In Australia and New Zealand, authorities have set a maximum level of 5 µg/kg phomopsins in lupine seeds in an effort to keep exposure as low as possible (ANZFA 1999). Only a few surveys for PHOA occurrence have been published so far, confirming PHOA occurrence for example in lupin seeds (Wood and Petterson 1986; Petterson et al. 1985; Wood et al. 1987; Than et al. 1994). In addition, in vitro inoculation experiments under unfavourable conditions have led to high PHOA concentrations in further grain legumes, including peas (Schloß et al. 2015b).

OTA is a widely known nephrotoxin, carcinogen, and weakly genotoxic (EFSA 2020). Its occurrence is well documented as storage toxin in grain commodities, including grain legumes.

In the majority of studies reporting on mycotoxin occurrence in plant-based commodities such as grain legumes, the forming fungus has not been elucidated (Kunz et al. 2020; Gruber-Dorninger et al. 2019; Woo et al. 2019; Ahn et al. 2016; Kolakowski et al. 2016; BVL 2015; Warth et al. 2012; Fakoor Janati et al. 2011; Kononenko and Burkin 2008; Beg et al. 2006; Valenta et al. 2002; Rafai et al. 2000; Scudamore et al. 1997). OTA is a known storage contaminant and also for PHOA, the toxin formation as a result of saprophytic spoilage seems to be relevant. However, the storage conditions as well as the distinct mycotoxigenic fungal strain strongly affect the extent of toxin formation. Especially, substrate, temperature, humidity and thus, water activity (*a*_*w*_) of the substrate play a significant role for growth and competitiveness (Beuchat, 1983; Magan and Lacey 1984) and toxin production (Moss 1991) of various fungal species.

OTA-producing fungi prolific during grain and grain legume storage include *Aspergillus* section *Circumdati* and Penicillium species, e.g., *Penicillium verrucosum*. As a member of the former, *Aspergillus westerdijkiae* has the potential to form large amounts of OTA in grains with OTA concentration as high as 8.45 g/kg after 21 days of inoculation (Ramos et al. 1998) and was subsequently chosen as a model organism. *A. westerdijkiae* was formerly assigned to *Aspergillus ochraceus* (Frisvad et al. 2004). The saprophytic fungus grows and produces OTA on multiple substrates: cereal grains (Aldred et al. 2008; Ramos et al. 1998; Pardo et al. 2004), fruit (Marino et al. 2009), coffee (Akbar et al. 2020; Einloft et al. 2017; Gil-Serna et al. 2015), and dried meat products such as ham and salami (Iacumin et al. 2020; Vipotnik et al. 2017; Meftah et al. 2018; Parussolo et al. 2019). Even though growth and OTA production of this fungus was not investigated in grain legumes yet, *Aspergillus* section *Circumdati* are present in grain legumes samples and products thereof. While one study specifically identified *A. westerdijkiae* on beans from Brazil (dos Santos-Ciscon et al. 2019), studies of the mycoflora of peas (*Pisum sativum*), lima beans (*Phaseolus lunatus*), broad beans (*Vicia faba*) (Hitokoto et al. 1981), peas and haricot beans (*Phaseolus vulgaris*) (Munimbazi and Bullerman 1996), and soybeans (*Glycine max*) (Mislivec and Bruce 1977) identified *A. ochraceus*, which was formerly indistinguishable from *A. westerdijkiae*. Phomopsins, including PHOA, are produced by *Diaporthe toxica*, formerly assigned as toxigenic *Phomopsis leptostromiformis* (Williamson et al. 1994). This fungus was identified as causing stem blight disease in *Lupinus* spp. (Ostazeski and Wells 1960; Ali et al. 1982). According to Shivas et al. (1991), isolates of the species previously described as *P. leptostromiformis* differ in their production of phomopsin A and C. Isolates that do not produce phomopsins have been reclassified to the species *Diaporthe woodii* (Williamson et al. 1994).

The two species *D. toxica* and *Diaporthe woodii* differ in their nucleotide sequences of the internal spacer region of the nuclear of the ribosomal RNA gene operon, the genes of the large subunit ribosomal RNA, histone H3, translation elongation factor 1-α and β-tubulin (Gao et al. 2017). Originally, the asexual state, anamorph, of both species was described as *P. leptostromiformis* (Williamson et al. 1994). To avoid taxonomic ambiguity, we use the species name *D. toxica* and *P. leptostromiformis* in this report according to the information by the supplying strain collection.

*P. leptostromiformis* was also isolated from clover (*Trifolium subterraneum*) (Shivas et al. 1991)*,* and might not be host specific. Nonetheless, so far, infection of seeds has only been observed in lupin plants (ANZFA 1999). In prior storage experiments by Allen et al. (1984) on lupine seeds after harvest, no increase in *P. leptostromiformis* infection rate and toxicity could be found, indicating that the conditions used were not appropriate to promote toxin production. However, *P. leptostromiformis* produced PHOA during storage experiments on further grain legumes such as peas, in vitro (Schloß et al. 2015b). It is expectable for grain legumes that they come in contact with other batches of grains, for instance, in common storage facilities. Thus, a spread of a saprophytic infection is possible.

The aim of the present study was to evaluate the impact of water activity and accompanying mycoflora on the formation of two important mycotoxins, PHOA and OTA, on peas, as model legumes under simulated storage conditions. Peas as grain legumes have been previously demonstrated to be suitable saprophytic hosts for *D. toxica*, a known producer of PHOA. As grain legumes, they are also susceptible to OTA contamination in storage. *D. toxica* was chosen as PHOA producing fungus in lupines. *A. westerdijkiae* was chosen as a common OTA producer of *Aspergillus* section *Circumdati* that has shown a high OTA production potential. For the present study, a realistic storage temperature of 20 °C was chosen. The samples were inoculated with the fungal strains and incubated for 14 days to assess both the fungal growth and the mycotoxin concentration along the duration. Intensity of toxin formation and growth will be monitored under different humidity conditions in order to evaluate the impact of unfavourable storage conditions.

We expected higher growth and mycotoxin production at higher water activities. Furthermore, we expected the pea mycoflora to compete and have an inhibitory effect on both growth and toxin production during cultivation. Thus, the mycoflora was also investigated and co-incubation experiments with *P. leptostromiformis* were conducted.

## Materials and methods

### Standards and chemicals

Ochratoxin A (OTA) was purchased as certified reference standard solution in acetonitril (ACN) (10.05 ± 0.8 μg/mL from solid standard at 99.5 ± 0.5%, concentration confirmed by the manufacturer via HPLC-FLD) from Romer Labs Division Holding GmbH (Getzersdorf, Austria). Phomopsin A (PHOA ≥ 98%) was purchased as solid substance from Biomol GmbH (Hamburg, Germany), dissolved in methanol, transferred, and dried under nitrogen stream in a pre-weighed vial and the determined weight dissolved in a known volume of methanol containing 6% formic acid. Isotopically labeled (d_5_-)OTA was purchased from LGC Standards GmbH (Wesel, Germany). ^15^N_6_-PHOA was isolated as a crude extract by preparative LC from liquid cultures of *P. leptostromiformis* that only received isotopically labeled nitrogen sources during growth according to Schloß et al. (2015a). In an LC–MS/MS measurement of the extract, no signal of remaining native ^14^N_6_-PHOA was found, proving the applicability as internal standard (IS).

ACN and methanol of LC–MS grade, formic acid (ACS reagent, reg. Ph. Eur.), and anhydrous MgSO4 (ReagentPlus®) were purchased from Merck KGaA (Darmstadt, Germany). For preparing doubly deionized water, a water purification system (Milli-Q® Reference A + System, Merck KGaA, Darmstadt, Germany) was used.

Whole dry field peas (*Pisum sativum* L.) of the variety Salamanca in a 25-kg bag were provided by Norddeutsche Pflanzenzucht Hans-Georg Lembke KG, Holtsee, Germany. It was chosen as a widely used variety of field peas.

Glucose, chloramphenicol, norfloxacin, and dichloran for microbiological media were purchased from Sigma-Aldrich Chemie GmbH (Taufkirchen, Germany). Dichloran Rose Bengal Chloramphenicol (DRBC) agar plates, potato dextrose agar, and maximum recovery diluent were bought from Oxoid Deutschland GmbH (Wesel, Germany).

For the PCR assay, primers were synthesized by Eurofins Genomics Germany GmbH (Ebersberg, Germany). Nucleoside triphosphates (dNTPs) were obtained from Promega GmbH (Walldorf, Germany) and cloned *Thermus aquaticus* DNA polymerase was purchased from VWR International GmbH (Darmstadt, Germany).

### Strains and their cultivation

The *Diaporthe toxica* strains CBS 534.93, CBS 535.93, and CBS 546.93 were obtained from the culture collection of the *Westerdijk Fungal Biodiversity Institute* (Utrecht, Netherlands). *Phomopsis leptostromiformis* DSM 1894 was bought from the *Leibniz Institute German Collection of Microorganisms and Cell Cultures* (DMSZ) GmbH (Braunschweig, Germany). The OTA-producing strain *Aspergillus westerdijkiae* MUCL 39539 (synonym NRRL 3174) was purchased from the *Belgian Co-ordinated Collections of Micro-organisms/Mycothèque de l'Université catholique de Louvain* (BCCM/MUCL, Louvain-la-Neuve, Belgium).

*Diaporthe* strains were grown in lupine flour-glucose broth developed for this study as there is no literature on *Diaporthe* cultivation in bouillon. To prepare this broth, 30 g of organic lupine flour were boiled with 200 mL of demineralized water for 10 min. This suspension was centrifuged for 5 min at 2000 × *g* and 18 °C. The supernatant was mixed with 20 g D-glucose and 100 mg chloramphenicol, filled up to 1 L with demineralised water and adjusted to a pH value of 5.6. This broth was autoclaved for 15 min at 121 °C and a pressure of 3 atm. Four milligrams norfloxacin were added sterile to 1 L of the warm broth.

The inoculated broth was incubated under continuous rotation at 40 rpm for 7 days at room temperature. Afterwards, the turbid, sporulated culture was removed under the filamentous growth. This suspension was centrifuged for 3 min at 4000 × *g* and 4 °C. The cell pellet of *Diaporthe* spores was suspended in 6 mL sterile physiological saline.

After a 5-day incubation period at 25 °C, the spores of *A. westerdijkiae* MUCL 39539 were washed from two Dichloran Rose Bengal Chloramphenicol (DRBC) agar plates with 2 mL maximum resuscitation solution and centrifuged for 3 min at 10,000 × *g*/min and 4 °C. The precipitate was resuspended in 1 mL sterile physiological saline.

### Inoculation of pea samples and enumeration

Portions of 10 g of autoclaved and as part of the autoclave program dried peas (at an *a*_*w*_ value of approximately 0.57) were aseptically filled into sterile vials. Each portion of peas was soaked with either 1 mL or 5 mL demineralized water, resulting in an *a*_*w*_ value of 0.94 or 0.98, respectively. Three hundred microliters from the spore suspensions of either *P. leptostromiformis*, the three *D. toxica* strains or the *A. westerdijkiae* strain were added and the inoculated peas were shaken for 1 min. One milliliter of spore suspension for inoculation contained approximately 10,000 CFU of fungal spores. The initial numbers with which the autoclaved peas were co-incubated by pure cultures *P. leptostromiformis* DSM 1894 and the moulds from non-autoclaved peas can be seen in the results section. The enumeration of *Aspergillus* spp. from inoculated peas was performed on Dichloran Rose Bengal Chloramphenicol (DRBC) agar. *Diaporthe* spp. were counted on potato extract glucose agar supplemented with 100 mg chloramphenicol, 4 mg norfloxacin and 4 mg dichloran per liter (modified PDA). The cultures were incubated at 20 °C at either 30%, 50%, or 80% relative air humidity inside a climate chamber. After 0, 1, 3, 7, and 14 days, one portion of peas was taken for counting of colony forming units (CFU). For this purpose, 1 ml sterile maximum recovery diluent was added to each ten-gram portion of inoculated peas. The wetted pea portions were subsequently shaken for one minute. A serial decimal dilution of the drained suspension with maximum recovery diluent was performed. One hundred microliters of these dilutions was plated on DRBC or modified potato-dextrose agar (PDA), respectively, and counted after incubation at 25 °C for 5 days. Two other portions of peas at each set of conditions were autoclaved and stored at – 20 °C for mycotoxin analysis.

### Identification of the natural microflora of dry peas

Fungi naturally occurring in dry peas were identified by PCR sequencing fragments of their calmodulin and β-tubulin genes according to Hong et al. (2005) and Glass and Donaldson (1995), respectively. Primers cmd5 and cmd6 from Hong et al. (2005) and Bt2a and Bt2b from Glass and Donaldson (1995) were applied, respectively. After amplification of the gene fragments, they were sequenced by Eurofins Genomics Germany GmbH (Ebersberg, Germany). Sequence homologies of the amplification products were compared from GenBank, accessed at 27.02.2021, using the BLAST program package (Altschul et al. 1997).

Accompanying bacteria of dry peas were identified after cultivation on a standard plate count agar (Becton Dickinson GmbH, Heidelberg, Germany) with microflex LT/SH matrix-assisted laser desorption ionization-time of flight mass spectrometer (MALDI-TOF MS; Bruker Daltonik GmbH, Bremen, Germany) and the MALDI Biotyper® database version 4.1.60 (Bruker Daltonik GmbH, Bremen, Germany).

### LC–MS/MS quantification of OTA and PHOA

The method described in Kunz et al. (2021) was applied with some minor changes: For OTA determination, the evaporation step of the sample extraction was omitted. For both toxins, the range of the external calibration was changed to broader ranges of 4.12 µg/kg to 412 µg/kg OTA and 5.94 µg/kg to 594 µg/kg PHOA.

### Standard solutions

For external calibration, mixes of OTA and PHOA stock solutions with their respective internal standard mix were prepared, resulting in a calibration line of eight equidistant data points. Concentrations ranged from 0.51 to 51.5 ng/mL (OTA) and 4.83 to 483 ng/mL (PHOA) with an approximate concentration of 5.15 ng/mL d_5_-OTA and 48.3 ng/mL ^15^N_6_-PHOA.

### Extraction procedure

Pea samples were frozen at – 25 °C, freeze dried (Delta 2–24 LSCplus, Martin Christ GmbH, Osterode am Harz, Germany) and milled in a centrifugal mill (ZM 200, Retsch GmbH, Haan, Germany) at 18,000 rpm with a 0.5-mm distance sieve. Of each of two biological replicates, three samples of each 2.5 g (or 1.0 g when the total material was not enough) were transferred into 50 mL centrifugation tubes. Extraction with 8 mL ACN with 0.1% formic acid and 2 mL doubly deionized water by shaking for 30 min (Multi Reax, Heidolph Instruments GmbH & Co.KG, Schwabach) followed. After centrifuging at 5800 × *g* for 30 min, 7.5 mL of the supernatant was transferred into a 15-mL centrifugation tube. For PHOA analysis, this supernatant was evaporated to dryness at 40 °C for 6 h at 10 mbar (RVC 2–33 Infrared rotation vacuum concentrator with condensation trap Alpha 2–4 LD plus, Martin Christ Gefriertrocknungsanlagen GmbH, Osterode am Harz, Germany). The residue was re-dissolved in 800 µL ACN with 0.1% formic acid and 200 µL doubly deionized water and shaken for 10 min (Multi Reax, Heidolph Instruments GmbH & Co. KG, Schwabach). For samples exceeding the calibration area, dilutions were prepared with a mix (8/2) of ACN 0.1% formic acid and doubly deionized water. Five hundred microliters of either the supernatant, the concentrated supernatant or dilutions thereof were transferred into a 2-mL-centrifuge tube, as well as 100 µL of an internal standard (IS) solution and 400 µL of saturated MgSO_4_ solution (approximately 333 g waterless MgSO_4_ per liter water) were added. After vortexing for 30 s, centrifuging at 17,000 × *g* at 10 °C for 10 min facilitated phase separation. Three hundred microliters of the upper organic layer was mixed with 300 µL doubly deionized water in a vial and stored at + 3 °C until measurement. Turbid samples were additionally passed through 0.45 µm nylon syringe filters.

### Instrumentation

Duplicate injections were performed for each vial. For chromatographic separation, a Shimadzu HPLC system (NEXERA X2, Shimadzu Deutschland GmbH, Duisburg, Germany) with a gradient program of eluent A water and eluent B methanol with each 300 mg/L ammonium formate and 0.1% formic acid was used: 0 min 15% B, 0.8 min 15% B, 4.0 min 60% B, 6.0 min 65% B, 8.5 min 80% B, 11.0 min 95% B, 12.0 min 95% B, 12.5 min 15% B, 15 min 15% B. The analytical column was a polyether ether ketone (PEEK)-coated polar C18 analytical column, 100 × 2.1 mm, 5 µm (ProteCol®, BGB Analytik Vertrieb GmbH, Rheinfelden, Germany) at a column oven temperature of 40 °C. Analyte detection was conducted with a triple quadrupole mass spectrometer (QTRAP 6500 + , Sciex Germany GmbH, Darmstadt, Germany) via multi reaction monitoring (MRM) in positive and negative electrospray ionization (ESI) modes. Curtain gas 40, CAD medium, temperature 300 °C, the ± ion spray voltage 4500 V, GS1 60, GS2 35, and varying dwell-time. Mass transitions, collision energies, and further parameters are given in Table 1.

**Table 1:** Mass transitions and conditions for LC–MS/MS quantification

Analyte	Retention time [min]	Precursor ion [m/z]	Measured ion	Product ions [m/z]	Declustering potential (DP) [V]	Collision energy (CE) [eV]	Collision cell exit potential (CXP) [V]
PHOA	4.02	789.3	PHOA + H^+^	323	66	35	18
				225.9		49	12
^15^N_6_-PHOA	4.02	795.3	^15^N_6_-PHOA + H^+^	227	66	49	12
OTA	6.72	404.1	OTA + H^+^	239	26	31	12
				357.9		19	18
d_5_-OTA	6.68	409.1	d_5_-OTA + H^+^	362.8	26	19	18

### Method evaluation and validation

Data evaluation was performed with MultiQuant Software, V. 3.0.2, AB Sciex Germany GmbH, Darmstadt, Germany.

Details on method validation are described in Kunz et al. (2021). Method performance data are given in Table 2.

**Table 2:** Method performance for PHOA and OTA quantification

**Analyte**	**LOD**^a^ **[µg/kg]**	**Limit of quantification** **[µg/kg]**	**Intraday precision** **[%]**	**Recovery** **[%]**	**Lowest point of external calibration** **[µg/kg]**	**Highest point of external calibration** **[µg/kg]**	**Correlation coefficient (*r*)**
Phomopsin A (PHOA)	1.68^b^	5.54^b^	5.6^b^	95.1^b^	5.94	594	0.999^b^
Ochratoxin A (OTA)	1.25^c^	4.11^c^	4.3^c^	104.7^c^	4.12	412	0.998^c^

^a^*LOD* limit of detection

^b^Data from Kunz et al. (2021)

^c^Data not published

## Results

### First incubation experiments without prior autoclavation of the whole peas

Prior to the incubation experiments on autoclaved peas under controlled microbial conditions, it was tested if *Phomopsis leptostromiformis* DSM 1894 and *Aspergillus westerdijkiae* MUCL 39539 would grow on pea material without prior autoclaving. Three biological replicates were incubated at 25 °C and 85% relative air humidity and an *a*_*w*_ value of 0.98. Thereby, numbers of the *P. leptostromiformis* strain decreased from 0.6 to 1 × 10^4^ CFU/g peas on day 0 to below 1 × 10^2^ CFU/g after 14 days of incubation. In contrast, the *A. westerdijkiae* strain grew to 0.8 to 1.2 × 10^6^ CFU/g peas in the same period, storage conditions, and initial numbers.

In day 0 and day 14 samples, OTA and PHOA concentration was measured. Neither on day 0 nor day 14, PHOA could be detected, while OTA content exceeded the calibration range of up to 37.8 µg/kg OTA. After dilution of one sample each, the OTA content in the two biological replicates was estimated as approximately 100 µg/kg and 1.2 mg/kg, respectively.

### Identification of the pea’s microflora

Non-autoclaved peas contained less than 10 CFU of fungi per gram and between 15 and 50 CFU of bacteria per gram. The bacteria were identified as the aerobic spore-formers *Bacillus pumilus* and *Bacillus muralis*. The fungi detected turned out to be the *Aspergillus* species *A. montevidensis* and *A. pseudoglaucus*.

### Fungal growth and mycotoxin formation under controlled conditions

Fungal microflora is highly diverse depending on the type of raw material and place of origin. The present study was carried out using autoclaved peas as experimental matrix in order to exclude the influence of any accompanying microorganisms on toxin formation and fungal growth.

**Fig. 2:**
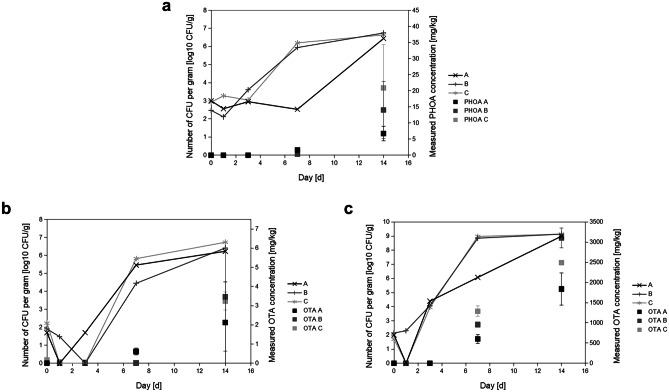
Numbers and measured mycotoxin concentration in peas inoculated at 20 °C. Colony forming units per gram are plotted on a logarithmic scale (left *y*-axis) and measured PHOA concentration in µg/kg on a linear scale (right *y*-axis). Error bars span to each of the two replicate cultures of each strain at each relative air humidity that were incubated at the same time. **a** *P. leptostromiformis* DSM 1894 at *a*_*w*_ 0.98. **b** *A. westerdijkiae* MUCL 39539 at *a*_*w*_ 0.94. **c** *A. westerdijkiae* MUCL 39539 at *aw* 0.98. Legend abbreviations: A 30% relative air humidity, B 50% relative air humidity, and C 80% relative air humidity

As visualized in Fig. 2, the increase in numbers of the inoculated fungal strains started around day 3 and slowed down at around day 7 in both *P. leptostromiformis* cultures at *a*_*w*_ 0.98 and *A. westerdijkiae* cultures at both *a*_*w*_ 0.94 and 0.98 Additionally, PHOA and OTA concentration exceeded the respective limit of detection (LOD) at day 7. Day 14 mycotoxin concentrations are also, on average, higher than day 7 mycotoxin concentrations.

For *P. leptostromiformis* DSM 1894, neither an increase in numbers over time nor a detectable mycotoxin level was reached at an *a*_*w*_ value of 0.94 until day 14 of incubation.

At an *a*_*w*_ value of 0.98, growth was observable both numerically and visually: the peas were indented, stuck together and showed black discolouration. During days 1 to 3 of the incubation, no PHOA was detectable (LOD 1.68 µg/kg). Starting on day 7, PHOA concentration ranged from 204 µg/kg to 1.85 mg/kg. On day 14, PHOA content ranged from 4.49 to 34.3 mg/kg, while the concentrations varied widely throughout the different air humidities. From the seventh to the fourteenth day of storage, the numbers of *P. leptostromiformis* DSM 1894 increased from 340 to 1.6 × 10^6^ CFU/g to 2.8 × 10^6^ to 5.6 × 10^6^ CFU/g.

For *A. westerdijkiae* MUCL 39539, OTA concentration for all three levels of relative air humidity (30%, 50%, 80%) on day 7 ranged from below LOD (1.25 µg/kg) to 781 µg/kg at an *a*_*w*_ value of 0.94 and 488 mg/kg to 1.42 g/kg at an *a*_*w*_ value of 0.98. Already at this point of time, at an *a*_*w*_ value of 0.98, OTA production was several order of magnitude higher than at an *a*_*w*_ value of 0.94. For both *a*_*w*_ levels, numbers at day 7 jumped up to 2.8 × 10^4^ to 6.6 × 10^5^ CFU/g (*a*_*w*_ 0.94) and 1.2 × 10^6^ to 9.6 × 10^8^ CFU/g (*a*_*w*_ 0.98). For the latter, fungal growth was clearly visible at this stage. On day 14, OTA concentration showed similar high differences between the two water activities of 4.56 µg/kg to 6.29 mg/kg at an *a*_*w*_ value of 0.94 and 1.44 to 3.35 g/kg at an *a*_*w*_ value of 0.98. From day 7 to day 14, both cultures increased their microbial numbers to 1.7 × 10^6^ to 5.4 × 10^6^ CFU/g for an *a*_*w*_ value of 0.94 and to 1.0 × 10^9^ to 1.4 × 10^9^ CFU/g for an *a*_*w*_ value of 0.98. This increase was, in most cases, lower than from day 3 to day 7 on a logarithmic scale, which is visible in the flattening of the curves between day 7 and day 14 in Fig. 2.

For an *a*_*w*_ value of 0.98, on day 14, peas were visibly covered by fungal growth, discoloured, dented, and sticking together.

Several decreasing quantifiable OTA concentrations ranging from levels around 10 up to 230 µg/kg in an extreme case were found in 0–3 day samples of *A. westerdijkiae* MUCL 39,539 as well as *P. leptostromiformis* DSM 1894 at 30% relative air humidity. As the samples were milled in succession, this suggests that analyte carry-over (not to be confused with carry-over of toxins from feed to animal products) was not entirely avoided, leading to false-positive results. However, those moderate OTA concentrations should have no influence on the interpretability of data from day 7 and day 14 at an *a*_*w*_ value of 0.98 as the concentrations of analyte carry-over are negligible in these samples compared to these values. For an*a*_*w*_ value of 0.94 at 80% air humidity where the highest carry-over values occurred, the day 14 samples contained OTA concentrations being more than 10 times higher.

### Characterization of *D. toxica* PHOA production

**Fig. 3:**
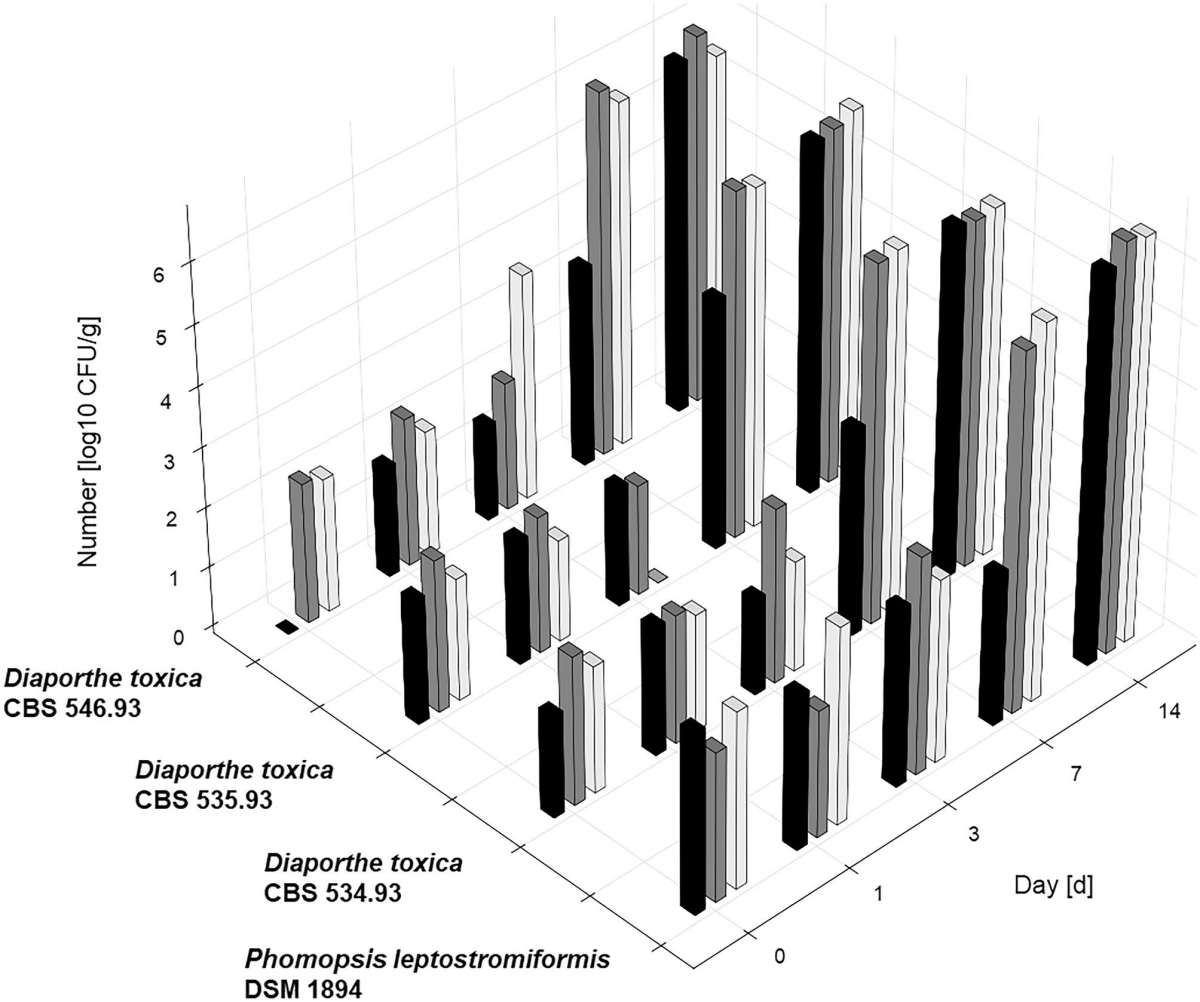
Colony forming units per gram of *P. leptostromiformis* DSM 1894 and three *D. toxica* strains in inoculated peas at 20 °C and an *a*_*w*_ value of 0.98 in a 3D bar chart, relative air humidities (black 30%, dark grey 50%, light grey 80%) grouped by incubation day

In addition to the detailed investigation of PHOA formation using *P. leptostromiformis* DSM 1894, three *D. toxica* strains were tested in a shortened protocol (14 days, 50% relative air humidity and an *a*_*w*_ value of 0.98) in duplicate, in order to monitor their toxin formation for comparison. All six samples showed white discolouration and fungal growth as well as indentation and the peas being stuck together. The three different *D. toxica* strains showed similar numbers [CFU/g] as the *P. leptostromiformis* strain during the course of the controlled storage (Fig. 3). Interestingly, all *Diaporthe* spp. strains consistently showed lower numbers at day 7 for 30% relative air humanity than for 50% and 80%. A comparison of the PHOA concentration in those *D. toxica* samples and the *P. leptostromiformis* samples at the same storage conditions (14 days, *a*_*w*_ value of 0.98 and 50% relative air humidity) is given in Table 3. The PHOA concentration in the *D. toxica* samples at 50% relative air humidity ranged from 28.3 to 32.4 mg/kg, thus with little deviation from the mean value. *P. leptostromiformis* incubations at all three relative air humidities (30%, 50%, and 80%) varied largely from 4.49 to 34.3 mg/kg. Independent of the humidity, the majority of PHOA concentrations (5 out of 6 values) determined at day 14 at an *a*_*w*_ value of 0.98 for *P. leptostromiformis* incubations were below the PHOA concentration determined for any of the *D. toxica* strains at 50% relative air humidity.

**Table 3:** PHOA content of peas inoculated with different strains of *Phomopsis leptostromiformis* and *D. toxica* as well as two co-incubations of *P. leptostromiformis* DSM 1894 with *Aspergillus montevidensis* and *A. pseudoglaucus* on day 14 of storage at 20 °C, 50% relative air humidity and an *a*_*w*_ value of 0.98

Strain	*Phomopsis leptostromiformis* DSM 1894		*Diaporthe toxica* CBS 534.93		*Diaporthe toxica* CBS 535.93		*Diaporthe toxica* CBS 546.93		*Phomopsis leptostromiformis* DSM 1894 with *Aspergillus montevidensis*		*Phomopsis leptostromiformis* DSM 1894 with *Aspergillus pseudoglaucus*	
Biological replicate	1	2	1	2	1	2	1	2	1	2	1	2
PHOA content [mg/kg]	22.9	5.15	28.3	29	32.4	29.5	32.1	29.4	8.04	6.48	33.9	50.3
Standard deviation [%]	1.6	2.2	3.0	2.6	2.1	3.2	3.5	2.2	2.4	3.1	1.9	4.4

### Influence of the microflora on the fungal growth of *P. leptostromiformis*

The spore-forming bacilli of the natural microflora did not affect the growth of *P. leptostromiformis* DSM 1894 on co-inoculated and previously autoclaved peas. On day 14 of storage at 20 °C, 50% relative humidity and an *a*_*w*_ value of 0.98, *P. leptostromiformis* DSM 1894 had grown to 1 to 4 × 10^7^ CFU/g, with and without the addition of 100 CFU *B. muralis* or *B. pumilus* per gram of peas.

From the fungal microflora identified on the unautoclaved pea material used in the present study, *A. montevidensis* and *A. pseudoglaucus* were chosen to be co-incubated with *P. leptostromiformis* DSM 1894. In the co-incubated peas with initially 3.7 × 10^2^ to 5.3 × 10^2^ CFU *P. leptostromiformis* DSM 1894/g, its number decreased slightly to 1 × 10^2^ to 4 × 10^2^ CFU/g after fourteen days of storage at 20 °C, 50% relative humidity and an *a*_*w*_ value of 0.98. In contrast, the two aspergilli in the same peas grew from an initial 10 CFU/g to 6 × 10^3^ to 8.7 × 10^4^ CFU/g.

PHOA concentration in the co-culture of *P. leptostromiformis* DSM 1894 and *A. montevidensis* was lower compared to *P. leptostromiformis* incubation at 50% relative air humidity and at an *a*_*w*_ value of 0.98 (8.04 and 6.48 mg/kg compared to 22.9 and 5.15 mg/kg). Conversely, PHOA production was higher when co-incubated with *A. pseudoglaucus* (33.9 and 50.3 mg/kg compared to 22.9 and 5.15 mg/kg; or 4.49 to 34.3 mg/kg at all levels of relative air humidity) (see Table 3).

## Discussion

The main goal of the present study was to clarify how far water activity and humidity influence growth as well as PHOA and OTA formation in dry field peas over time. Growth for *Phomopsis leptostromiformis* DSM 1894 was only detected at higher water activity. Fungal growth and OTA production for *Aspergillus westerdijkiae* MUCL 39539 were greatly enhanced by the higher water activity. The results of the study confirmed the high impact of water activity as a main factor for enhanced fungal growth of various saprophytic fungal species (Beuchat 1983; Magan and Lacey 1984). Various studies have similarly shown dependence of OTA production on water activity (Harwig and Chen 1974; Ramos et al. 1998; Pardo et al. 2004; Cairns-Fuller et al. 2005; Gil-Serna et al. 2015), which could be confirmed in the present study. To the authors’ knowledge, the present study is the first to investigate the relationship between relative air humidity, water activity, and PHOA production for *Diaporthe* spp.

In contrast, the relative air humidity (tested at 30%, 50%, or 80%) only had an effect on day 7 at 30% relative air humidity: Lower numbers were observed at 30% relative air humidity than at the two higher air humidities for *P. leptostromiformis* DSM 1984, *D. toxica* strains, and *A. westerdijkiae* MUCL 39539 at an *a*_*w*_ value of 0.98.

### Water activity and typical storage conditions of dry field peas

Water activity in the pea portions inoculated in the present study was measured to be at 0.94 for 1 mL water addition and at 0.98 for 5 mL water addition to originally dry seed quality material. According to Pixton and Henderson’s (1979) data on desorption and adsorption isotherms in dried peas, a moisture content of more than 24% would be required to reach these water activity values, indicating that moisture content and water activity in the present study is much higher than in dry whole peas as Gane (1948) describes them. The initial water content of whole peas was 13.3%. At 10°C, the moisture content was 9.0% at 30% relative air humidity, 12.3% at 50% and 17.2% at 80% relative air humidity (Gane 1948).

Autoclavation in the present study not only inactivated the co-occurring microflora but may have changed the pea structure to facilitate hyphen growth. Fungal spores were added in a large amount on purpose. In addition, water was added to raise moisture content. Dadgar (2005) showed that whole field peas without former water addition, autoclavation, or spore solution addition at an initial moisture content of 10.35% only developed mould at relative air humidities of 80% and above—the samples that spoiled the fastest still took 18 days, so much longer than in the present study, to show visible fungal infection. The observed fungi were assigned to *Aspergillus* spp. or *Penicillium* spp. Mills and Woods (1994) suggested diagrams of moisture content and temperature and when ‘safe storage’ of field peas without off-odour or mould development is possible by combining data from 5 m diameter metal bins and lab experiments. For a temperature of 20 °C, the safe zone is below 15% moisture content. The main fungal species growing under these conditions were *Erotium* spp., *Penicillium* spp. and, to a lower extent, *A. ochraceus*.

Storage conditions used in the present study reflect unfavourable conditions such as wet spots with higher moisture content that can be formed by temperature shifts (Pixton and Warburton 1971) and storage temperatures at the upper end of realistic conditions in temperate climate. Recommended pea moisture levels for transport and storage, where spoilage can be largely prevented, is considered 16.1% (Canadian Grain Commission 2018), 15% (USA Pulses 2021) and 14–16% (The German Insurance Association 1989–2021). As storage temperature, 15 °C is recommended (Alberta Pulse Growers 2021) or a range of 5–25 °C (The German Insurance Association 1989–2021).

### Growth and mycotoxin formation

Some of the numbers of *A. westerdijkiae* MUCL 39539 and *P. leptostromiformis* DSM 1894 show lower values on day 1 to 7 than on day 0 in Fig. 2 and additionally provided as tables in Online resource 1. These numbers were derived from ten-step serial dilutions of spore extracts from a single biological replicate each and are subject to biological differences. A big impact on the precision of the numbers is the homogeneity of the material. Whole peas are more inhomogeneous than flour but the study’s aim was to test a realistic setup with whole peas; thus, the lower precision was accepted.

### PHOA

This present study shows a lack of growth and toxin formation at an *a*_*w*_ value of 0.94, which suggests that higher water activities were needed for the fungus to grow and produce PHOA. For similar investigation of PHOA production during controlled storage, few studies are available. Allen et al. (1984) stored lupine seeds naturally infected with *P. leptostromiformis* either in a shed (1–46 °C and 12 to 82% relative air humidity) or in a controlled humid environment (25–27 °C and 70 to 90% relative air humidity). During the storage period of 45 weeks, several samples were taken and a liver toxicity assay on sheep was conducted according to Allen et al. (1978). The stored lupines samples did not show the tendency to grow in toxicity monitored as sheep liver damage. After the storage period, the level of infection (percentage of a sample of 400 seeds that showed *P. leptostromiformi*s growth in medium after surface disinfection) was even slightly reduced.

PHOA concentration ranged from 4.49 to 34.3 mg/kg after 14 days in the present study. Under unfavourable conditions (30 g seeds, 10 mL water addition, 4 weeks incubation at 24 °C), *P. leptostromiformis* produced PHOA *in vitro* in lupines as well as other grain legumes than lupines (Schloß et al. 2015b). The values ranged from approximately 220 mg/kg in grain lupines, 300 mg/kg in lupine plants, 320 mg/kg in white beans to 440 mg/kg in peas. The higher PHOA concentration as compared to the present study might be explained by a longer incubation period (twice as long as in the present study) and the temperature closer to the PHOA production optimum in liquid culture of 25 °C according to Lanigan et al. (1979).

The present study does not find a direct correlation of growth and toxin formation as growth (measured by numbers [CFU/g]) declined around day 7, but PHOA production was still increasing. Both Lanigan et al. (1979) in liquid media, and Shankar et al. (1999) in lupine plant parts taken from latently infected plants, also found that PHOA production by *P. leptostromiformis*, similarly to *A. westerdijkiae*’s OTA production, did not correlate well with mycelium growth.

PHOA concentration of peas inoculated with *P. leptostromiformis* DSM 1894, at an *a*_*w*_ value of 0.98 and on day 14 ranged from 4.49 to 34.3 mg/kg, thus varied widely. Two values (22.9 and 34.3 mg/kg) were close to the PHOA concentration in the peas inoculated with *D. toxica* at 50% relative air humidity of approximately 30 mg/kg. Therefore, we assume the toxigenic strain *P. leptostromiformis* DSM 1894 belongs to the species *D. toxica*.

### OTA

In the present study, at an *a*_*w*_ value of 0.98, concentrations in the g/kg range are found after 14 days. Similar studies to the present work, describing growth of *A. westerdijkiae* NRRL 3174 on autoclaved wheat and barley led to the formation of OTA in the same range as the present study with 5.89 g/kg OTA after 14 days at 25 °C and at an *a*_*w*_ value of 0.98 (Ramos et al. 1998) as well as OTA in g/kg range with a strain depicted as *A. ochraceus* in shredded wheat (Harris and Mantle 2001).

The present study finds that OTA production of *Aspergillus westerdijkiae* MUCL 39539 was enhanced at *a*_*w*_ value of 0.98 compared to 0.94. The same tendency was observed for OTA production of two *A. westerdijkiae* strains (including CECT 2948, which is equivalent to NRRL 3174 and MUCL 39539, and an unspecified strain) in Czapek Yeast Extract Agars prepared from paprika, green coffee, anise, grapes, maize, and barley assessed by Gil-Serna et al. (2015). At an *a*_*w*_ value of 0.928, both *A. westerdijkiae* strain cultures showed lower OTA concentration than at *a*_*w*_ values of 0.964 and 0.995. Besides, the present study finds that the majority of toxin production started around day 7, while the growth rate decreased. Gil-Serna et al. (2015) also found that sporulation and growth could not be correlated with OTA production.

It is unclear which further environmental elicitors lead to enhanced OTA production and might be causative for differences between OTA-producing species (Wang et al. 2016). So, it remains unclear which exact conditions might have facilitated the high OTA levels in the range of g/kg in the present study at days 7 and 14 of incubation with an *a*_*w*_ value of 0.98.

### Influence of the microflora of peas on fungal growth

Under the initial experimental conditions (untreated moistened peas without autoclaving), repeated three times, the pea raw material contained a background microflora that might have prevented growth of *P. leptostromiformi*s DSM 1894 on the grain legumes whilst growth of *A. westerdijkiae* MUCL 39539 was less affected (0.8 to 1.2 × 10^6^ CFU/g on day 14 without autoclavation and 1.0 × 10^9^ to 1.4 × 10^9^ CFU/g on day 14 with autoclavation, both at an *a*_*w*_ value of 0.98). *A. montevidensis* and *A. pseudoglaucus* have been identified from the fungal microflora on the pea material. Both are known to produce bioactive secondary metabolites such as cladosporin and mycophenolic acid that might influence the growth of accompanying fungi (Greco et al. 2015; Mouhamadou et al. 2016). In the co-culture experiments, the two aspergilli of the natural pea microflora inhibited the growth of *P. leptostromiformis* DSM 1894.

Allen et al. (1984) also found in storage experiments with infected lupine seeds that after 45 weeks of storage in a controlled humid environment, 29.5% and 54.0% of the seeds showed an infection with *Aspergillus* spp. It is thus possible that the lack of growth of *P. leptostromiformis* in the humid environment was influenced by additional unidentified fungal members of the microflora on the seeds. The authors also hypothesized that the viability of the PHOA producer was first reduced (after 39 weeks) and eventually eliminated after 45 weeks by overgrowth with *Aspergillus* spp.

The PHOA production of *P. leptostromiformis* conversely even increased in the present study with an *A. pseudoglaucus* co-incubation. One possibility is that the fungus imposed additional stress on *P. leptostromiformis*, possibly leading to an excessive toxin pathway activation—but the activation pathway is not elucidated yet. An alternative explanation would be that the fungus enhanced PHOA production by providing precursors of the toxin biosynthesis without promoting growth. Lanigan et al. (1979) showed that *P. leptostromiformis’* growth and PHOA production was limited by peptide availability. Proteolytic activity from *A. pseudoglaucus* that produces proteases and can be used for fermentation purposes (Liu et al. 2018) might thus lead to a higher peptide concentration and consequently enhance PHOA production. The co-culture experiment was conducted under controlled conditions with autoclaved peas at 20 °C, 50% relative humidity, an *a*_*w*_ value of 0.98 and defined low initial fungal counts.

On the other hand, the reduced PHOA formation in the co-culture with *A. montevidensis* is remarkable. The potential for biocontrol of PHOA producers on host plants by non-toxicogenic *A. montevidensis* isolates should be investigated under further controlled incubation conditions.

In the present study, *A. westerdijkiae* MUCL 39539 OTA concentration in unautoclaved peas after 14 days ranged from approximately 100 µg/kg to approximately 1.2 mg/kg, which is much lower than the values for autoclaved peas that ranged between 1.44 g/kg and 3.35 g/kg—both at an *a*_*w*_ value of 0.98. Inhibition of OTA production through microflora has also been observed with both with fungal microflora (strain NRRL 3174; Chelack et al. 1991) and bacterial flora consisting of various *Bacillus* species (strain NRRL 3174 and another strain isolated from green coffee; Petchkongkaew 2008; Einloft et al. 2017).

It is noteworthy, though, that the amount of colony forming units on the non-autoclaved peas was less than 10 CFU of fungi per gram and between 15 and 50 CFU of bacteria per gram, thus very low.

*Diaporthe* spp. and *A. westerdijkiae* are all saprophytic fungi in grain legumes, yet both show different tolerance to the given storage conditions. Particularly for PHOA formation, controlling the water activity in grain legumes could be a promising approach to prevent the post-harvest mycotoxin contamination. However, the intense formation in a relatively short time span for both toxins makes it obvious that only small hotspots of toxin formation can be sufficient to spoil an entire grain legume batch. No specific cut-off value can be suggested to prevent mycotoxin contamination in general. Even short times of fungal growth under unfavourable conditions (e.g., hotspots in a silo) might result in toxin amounts that can render the entire lot in the silo unusable. Potentially co-occurring microorganisms could prevent the growth of toxigenic fungi. However, the distinct species and modes of action require further investigation.

## Supplementary information

Below is the link to the electronic supplementary material.

Supplementary file1 (PDF 99 KB)

## Abbreviations

OTA: Ochratoxin A
PHOA: Phomopsin A
*a*_*w*_: Water activity
ACN: Acetonitrile
CFU: Colony forming units
IS: Internal standard
LOD: Limit of detection
