# Supplementary material for: Growth and toxin production of phomopsin A and ochratoxin A forming fungi under different storage conditions in a pea (Pisum sativum) model system
Source: Mycotoxin Res. 2021 Dec 18;38(1):37–50. doi: 10.1007/s12550-021-00446-8 (PMC8816495; doi:10.1007/s12550-021-00446-8)
Supplement: Supplementary file 1 — Supplementary file1 (PDF 99 KB) [file 12550_2021_446_MOESM1_ESM.pdf]

Online resource 1 Numbers of *P. leptostromiformis* DSM 1894 and *A. westerdijkiae* MUCL 39539 inoculated peas at 20 °C and an a_w value of 0.94 or 0.98 at 30%, 50% and 80% relative air humidity, as well as the corresponding PHOA or OTA concentration, presented in table form.

Article title: Growth and toxin production of phomopsin A and ochratoxin A forming fungi under different storage conditions in a pea (*Pisum sativum*) model system.

Journal name: Mycotoxin Research.

Author names: Birgitta Maria Kunz, Laura Pförtner, Stefan Weigel, Sascha Rohn, Anselm Lehmacher, Ronald Maul*

*Affiliation and e-mail address of the corresponding author: German Federal Institute for Risk Assessment (BfR), Department for Safety in the Food Chain, Max-Dohrn-Str. 8-10, 10589 Berlin, Germany. Max Rubner-Institut, Federal Research Institute of Nutrition and Food, Department Safety and Quality of Milk and Fish Products, Hermann-Weigmann-Straße 1, 24103 Kiel, Germany. Ronald.Maul@mri.bund.de

Table S1 Numbers and PHOA content in peas inoculated with *Phomopsis leptostromiformis* DSM 1894 at an a_w value of 0.94

Day of incubation [d]	30% relative air humidity			50% relative air humidity			80% relative air humidity		
	Number [CFU/g]	PHOA content ± standard deviation (2 biological replicates) [µg/kg]		Number [CFU/g]	PHOA content ± standard deviation (2 biological replicates) [µg/kg]		Number [CFU/g]	PHOA content ± standard deviation (2 biological replicates) [µg/kg]	
0	360	n.d.	n.d.	10	n.d.	n.d.	720	n.d.	n.d.
1	100	Not determined	Not determined	36	Not determined	Not determined	400	Not determined	Not determined
3	50	Not determined	Not determined	12	Not determined	Not determined	180	Not determined	Not determined
7	$1 \cdot 10^3$	Not determined	Not determined	380	Not determined	Not determined	20	Not determined	Not determined
14	110	n.d.	n.d.	40	n.d.	n.d.	< 1	n.d.	n.d.

Table S2 Numbers and PHOA content in peas inoculated with *Phomopsis leptostromiformis* DSM 1894 at an a_w value of 0.98

Day of incubation [d]	30% relative air humidity			50% relative air humidity			80% relative air humidity		
	Number [CFU/g]	PHOA content ± standard deviation (2 biological replicates) [µg/kg]		Number [CFU/g]	PHOA content ± standard deviation (2 biological replicates) [µg/kg]		Number [CFU/g]	PHOA content ± standard deviation (2 biological replicates) [µg/kg]	
0	1×10^3	n.d.	n.d.	300	n.d.	n.d.	860	n.d.	n.d.
1	370	n.d	n.d.	130	n.d.	n.d.	1.9×10^3	n.d.	n.d.
3	900	n.d.	n.d.	4.1×10^3	n.d.	n.d.	1.1×10^3	n.d.	n.d.
7	340	1,849 ± 1.0%	1,366 ± 1.3%	8.8×10^5	421 ± 3.6%	204 ± 3.8%	1.6×10^6	1,488 ± 0.7%	1,644 ± 3.5%
14	$2,8 \times 10^6$	4,490 ± 2.5%	8,920 ± 0.8%	5.6×10^6	22,930 ± 1.6%	5,151 ± 2.2%	4.4×10^6	7,518 ± 2.7%	34,292 ± 3.4%

Table S3 Numbers and OTA content in peas inoculated with *Aspergillus westerdijkiae* MUCL 39539 at an a_w value of 0.94

Day of incubation [d]	30% relative air humidity			50% relative air humidity			80% relative air humidity		
	Number [CFU/g]	OTA content ± standard deviation (2 biological replicates) [µg/kg]		Number [CFU/g]	OTA content ± standard deviation (2 biological replicates) [µg/kg]		Number [CFU/g]	OTA content ± standard deviation (2 biological replicates) [µg/kg]	
0	50	n.d.	n.d.	80	15.6 ± 5.7%	n.d.	170	92.1 ± 11%	230 ± 10%
1	< 1	n.d.	n.d.	30	n.d.	< limit of quantification (4.11)	< 1	47.0 ± 3.6%	86.2 ± 2.0%
3	50	15.1 ± 4.2%	6.06 ± 8.5%	< 1	n.d.	9.02 ± 17%	< 1	20.6 ± 3.7%	64.6 ± 15%
7	2.9×10^5	440 ± 8.9%	781 ± 6.3%	2.8×10^4	n.d.	7.75 ± 12%	6.6×10^5	21.4 ± 10%	47.0 ± 5.5%
14	1.7×10^6	4.56 ± 22%	4,253 ± 2.4%	2.6×10^6	6,287 ± 1.6%	630 ± 6.0%	5.4×10^6	2,778 ± 3.1%	3,713 ± 2.1%

Table S4 Numbers and OTA content in peas inoculated with *Aspergillus westerdijkiae* MUCL 39539 at an a_w value of 0.98.

Day of incubation [d]	30% relative air humidity			50% relative air humidity			80% relative air humidity		
	Number [CFU/g]	OTA content ± standard deviation (2 biological replicates) [µg/kg]		Number [CFU/g]	OTA content ± standard deviation (2 biological replicates) [µg/kg]		Number [CFU/g]	OTA content ± standard deviation (2 biological replicates) [µg/kg]	
0	100	n.d.	< limit of quantification (4.11)	140	n.d.	9.89 ± 11%	50	11.8 ± 3.3%	47.4 ± 2.1%
1	< 1	n.d.	9.76 ± 17%	200	n.d.	6.73 ± 14%	< 1	11.3 ± 14%	41.0 ± 7.3%
3	2.5×10^4	4.65 ± 2.2%	10.2 ± 3.1%	1.2×10^4	n.d.	8.50 ± 4.2%	9×10^3	11.1 ± 2.4%	19.2 ± 10%
7	1.2×10^6	709,631 ± 0.4%	488,188 ± 5.8%	7.2×10^8	1,006,208 ± 5.1%	911,684 ± 1.0%	9.6×10^8	1,158,389 ± 6.1%	1,419,612 ± 4.6%
14	1.0×10^9	2,237,345 ± 1.4%	1,441,579 ± 3.7%	1.4×10^9	3,351,480 ± 2.0%	2,862,464 ± 0.9%	1.4×10^9	2,435,848 ± 0.4%	2,554,760 ± 1.3%
